# Transaldolase Deficiency in a Saudi Girl: Identification of a Novel Homozygous TALDO1 Variant

**DOI:** 10.7759/cureus.93294

**Published:** 2025-09-26

**Authors:** Khalid Asiri, Syed Rayees, Badriah G Alasmari

**Affiliations:** 1 Department of Pediatric Gastroenterology, Armed Forces Hospital Southern Region, Khamis Mushait, SAU; 2 Department of Pediatrics, Armed Forces Hospital Southern Region, Khamis Mushait, SAU

**Keywords:** autosomal recessive disease, novel mutation, pediatric genetics, taldo1, transaldolase

## Abstract

Transaldolase deficiency (TALDOD) is a rare autosomal recessive disorder that affects the pentose phosphate pathway, resulting from pathogenic variants in the TALDO1 gene. The condition leads to varied multisystem involvement, including hepatosplenomegaly, liver dysfunction, coagulopathy, cardiac anomalies, facial dysmorphic traits, and renal anomalies, often presenting in infancy or early childhood. We present a case of a nine-month-old Saudi girl with clinical features consistent with TALDOD. Whole exome sequencing confirmed the presence of a homozygous novel variant c.871_873delGAG p.(Glu291del) in exon 7 of the TALDO1 gene with isoform NM_006755.1. To our knowledge, this variant has not been reported in the literature to date. The objective of this case report is to raise awareness about this very rare disease and to add it to the database of TALDOD.

## Introduction

Transaldolase deficiency (TALDOD) is a disease characterized by very low levels of the transaldolase enzyme, thereby disrupting the pentose phosphate pathway. It is an autosomal recessive disease and one of the rare inborn errors of metabolism caused by a mutation in the TALDO1 gene on chromosome 11p15 [1]. It is presented as a multisystem, early-onset disease. The enzyme transaldolase plays a vital role in the non-oxidative pentose phosphate pathway, delivering ribose-5-phosphate for nucleic acid production and NADPH for lipid synthesis. In the absence of this enzyme, harmful intermediate substances can accumulate, leading to a range of clinical manifestations, which may include intrauterine growth retardation, hydrops fetalis, liver dysfunction, hepatosplenomegaly, anemia, thrombocytopenia, and dysmorphic characteristics (cutis laxa, low-set ears, and antimongoloid slant eyes) [2,3]. Patients may present at various stages: prenatally, exhibiting intrauterine growth restriction and/or oligohydramnios; during the neonatal period, characterized by dysmorphic facial features, cardiovascular anomalies, and hepato(spleno)megaly; or later in life, displaying a milder phenotype or potentially no symptoms at all [4]. The serum and urine contain abnormal levels of polyols and sugars [5,6]. However, the confirmatory test is conducted through a whole-exome sequencing study. There is no known cure for this disease, and management primarily focuses on alleviating symptoms. The prognosis for TALDOD varies significantly and is influenced by the onset and the intensity of symptoms.

## Case presentation

We report a case involving a nine-month-old girl who was referred to our hospital due to severe malnutrition, significant weight loss, hepatosplenomegaly, and congenital heart defects (multiple septal defects). She was delivered at term via spontaneous vaginal delivery, with a birth weight of 2.1 kg and intrauterine growth retardation during pregnancy. She did not require admission to the neonatal intensive care unit (NICU) and had an unremarkable neonatal course. Her parents were consanguineous.

On presentation, the patient appeared smaller than expected for her age, with a weight below the third centile on the growth chart, and exhibited dysmorphic features, including a triangular face, a small chin, and thin lips. A chest examination indicated clear breath sounds bilaterally, while cardiovascular auscultation detected a pansystolic murmur predominantly at the apex, accompanied by normal first and second heart sounds. The abdominal examination revealed a soft, non-tender abdomen with the liver palpated 5 cm below the costal margin and the spleen 6 cm below the costal margin, with no signs of ascites.

Initial investigations revealed normal reference ranges for complete blood count (CBC), coagulation profile, electrolytes, renal function tests, thyroid function tests, ammonia, and lactate levels, as well as elevated levels of alpha-fetoprotein and liver enzymes (Table 1).

**Table 1:** Laboratory findings INR: international normalized ratio, PT: prothrombin time, aPTT: activated partial thromboplastin time

Measured lab test	Result	Normal range
Aminotransferase (ALT)	100 U/L	11-39 U/L
Aspartate aminotransferase (AST)	140 U/L	22-58 U/L
Gamma-glutamyl transferase (GGT)	73 U/L	5-40 U/L
White blood cells	7 units 10^9^L	4.5-13.5 units 10^9^L
Platelets	200 units 10^9^/L	150-400 units 10^9^/L
Hemoglobin	11.1 g/L	10.9-15 g/L
INR	1.00	<1.2
PT	13.9	11.5-15
aPTT	30.5 sec	26-36
Alpha-fetoprotein	15 ng/mL	1.09-8.04 ng/mL
Aerum vitamin D 25(OH)	25 ng/dL	20-50 ng/mL
Direct bilirubin	10 umol/L	1.7-8.6 umol/L

**Figure 1:**
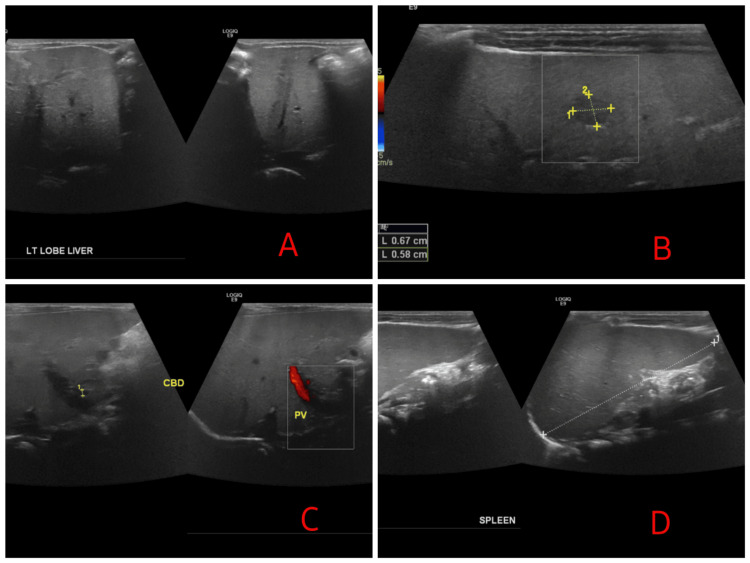
Ultrasound images of the (A) left lobe of the liver, (B) right lobe of the liver, (C) common bile duct, and (D) spleen (A, B) Enlarged liver, with a total span of 87 mm. (B) A hypoechoic lesion 7x5 mm (yellow dots) in the right lobe of the liver. (C) The common bile duct and patent portal vein. (D) Enlarged spleen with a span of 71 mm.

Regarding radiological investigations, an abdominal ultrasound (Figure 1) revealed an enlarged liver, with a span of 87 mm, a well-defined hypoechoic lesion measuring 7x5 mm in the right lobe, and an enlarged spleen with a span of 71 mm. A cardiac echo showed a moderate ventricular septal defect and a small atrial septal defect II. MRI Abdomen demonstrated hepatomegaly with fibrotic changes, splenomegaly, and a liver lesion in segment V, suggestive of mesenchymal hamartoma.

In view of the dysmorphic traits, hepatosplenomegaly, congenital heart disease, and failure to thrive, a whole exome sequencing (WES) was conducted (Table 2). It identified a homozygous novel variant c.871_873delGAG p.(Glu291del) in exon 7 of the TALDO1 gene with isoform NM_006755.1, confirming TALDOD. This is an in-frame deletion that leads to the deletion of one glutamic acid residue. This variant is classified as a variant of uncertain significance (VUS) and has not been reported in any case of TALDOD published so far.

**Table 2:** Whole exome sequencing

Gene (isoform)	OMIM-P	Variant	Zygosity	Variant classification
TALDO1 (NM_006755.1)	606003	c.871_873delGAG p.(Glu291del) chr11:754323	homozygous	Uncertain significance

The patient was managed supportively with ursodeoxycholic acid for cholestasis and a high-fat diet. She is currently undergoing follow-up with a multidisciplinary team consisting of metabolic medicine specialists, gastroenterologists, dietitians, geneticists, nephrologists, and cardiologists.

The patient is currently seven years of age and is not achieving adequate weight gain, although neurological assessments indicate normalcy. The concern regarding not gaining weight despite implementing various dietary strategies was brought to the attention of a dietitian, and a plan for inserting a gastrostomy tube was proposed; however, the family declined this option. She is being closely monitored for any potential complications. Her recent liver profile results are within normal limits. Ultrasound abdomen showed hepatomegaly with a persistent hypoechoic lesion in the right lobe of the liver, a patent portal vein of 8 mm, and an intrahepatic IVC with color flow noted at the visualized part of the hepatic veins, an unremarkable gall bladder, a CBD 2 mm, a right kidney with fullness of the extrarenal pelvis, an unremarkable left kidney, and a spleen span of 100 mm. Echocardiography showed a spontaneously closed VSD and ASD. Up to now, she has been compliant with her medication regimen and participates in quarterly outpatient follow-up appointments with access to the emergency department.

## Discussion

TALDOD, also known as Eyaid syndrome, derives its name from Dr. Eyaid, who published a case series in 2013 that included 12 patients from six Saudi families, confirming the disease's existence in conjunction with high consanguinity levels. The first documented case was presented in 2001 by Verhoeven et al., involving a patient who suffered from liver dysfunction shortly after birth and subsequently developed liver cirrhosis by the age of two [1]. By 2025, fewer than 50 patients have been reported in the literature, with most cases originating from the Mediterranean region. Reports have emerged from Saudi Arabia, the United Arab Emirates, Turkey, Poland, Amsterdam, and China. TALDO-D represents an inborn error of metabolism that impacts the pentose phosphate pathway. Elevated levels of polyol, heptulose, sedoheptulose, mannoheptulose, and sedoheptulose-7-phosphate are predominantly found in the urine and serum. The buildup of sugars and polyols, such as sedoheptulose-7-phosphate, ribose-5-phosphate, ribulose-5-phosphate, xylulose-5-phosphate, and C5-polyols (including D-ribitol and D-arabitol), is thought to contribute to liver complications in TALDOD [1,2]. TALDOD has an autosomal recessive mode of inheritance, highlighting the importance of consanguinity in these cases.

The primary symptoms in individuals with TALDOD include anaemia, bleeding complications associated with thrombocytopenia, hepatosplenomegaly, progressive nodular hepatic fibrosis, and, subsequently, nephropathy. Most of these patients have consanguineous parents. The early onset form is typically severe and often fatal, whereas the late onset form is less severe and progresses more slowly [2,3]. Liver dysfunction starts early in fetal development in both forms of presentation. In the early onset presentation, liver involvement typically manifests as coagulopathy, transaminitis, hypoalbuminemia, and skin changes in newborns. The primary factor contributing to reduced lifespan is progressive liver failure. In patients with a later onset of the disease, liver dysfunction is less severe and progresses at a slower rate [3,4]. Intrauterine growth restriction, hydrops fetalis, oligohydramnios, and neonatal edema are among the prenatal manifestations. Antenatally, dysmorphic features can be notably observed, including triangular facies, low-set ears, cutis laxa, and thin lips. A few patients who presented late with the disease experienced growth impairment. Other signs and symptoms that have been reported include abnormal external genitalia, proximal and distal renal tubular dysfunction, anemia, thrombocytopenia, pancytopenia, hepatomegaly, splenomegaly, hepatic dysfunction, and congenital heart defects, such as VSD and ASD [4,5].

Biochemical assessments of urinary sugars and polyols can be used to diagnose TALDOD; however, confirmation is typically achieved through molecular genetic testing. To date, effective treatment for TALDOD has not been established. Management entails the non-specific treatment of related defects, which consists of surgical correction of cardiac abnormalities, supportive symptomatic interventions including calcium and vitamin D supplementation, transfusions of red blood cells and platelets, and albumin therapy [5]. N-acetylcysteine, in conjunction with antioxidants such as vitamins C and E, is proposed to mitigate oxidative stress. The use of specific protein phosphatase (PPP) inhibitors is also advised to decrease polyol or sugar-P accumulation [6]. Liver transplantation performed following liver failure carries a minor risk of recurrence.

Timely and precise prenatal diagnosis can significantly improve outcomes and facilitate enhanced prenatal management, which includes strategies for fetal monitoring and suitable postpartum care. Notably, an increased frequency of targeted ultrasound fetal surveillance can assist in detecting early clinical signs (e.g., elevated MCA-PSV, cardiomegaly, and placental thickness), which serve as critical prognostic markers. Above all, this process is fundamentally reliant on the collaborative efforts of a multi-disciplinary team [6,7].

TALDO deficiency may be easily misidentified as gestational alloimmune liver disease (GALD). GALD is caused by maternal alloimmune injury, resulting in neonatal liver failure (which includes coagulation disorders, ascites, and hypoalbuminemia) and the accumulation of iron, both intrahepatic and extrahepatic (hemosiderosis) [7,8]. Research is presently underway to discover treatments for TALDOD. In 2019, a study was performed on the supplementation of N-acetyl cysteine (NAC) [9] to maintain consistent levels of alpha-fetoprotein. This could reflect a reduction in hepatocyte injury and a lower incidence of hepatocarcinogenesis, as proposed in the mouse disease model; however, further exploration is essential in this area.

## Conclusions

In conclusion, our analysis suggests that TALDOD is a pleiotropic disorder that must be considered when exploring cases in infancy involving unexplained hepatosplenomegaly, congenital heart disease, and failure to thrive, especially in consanguineous families. While there is presently no specific treatment available, focused molecular analysis of the TALDO1 gene in amniotic fluid or chorionic villi can provide significant assistance to affected families in making informed reproductive decisions. Genetic counseling is essential in the context of consanguineous marriage because of the autosomal recessive mode of inheritance of the disease. This case report aims to contribute to registries and improve diagnostic recognition.

## References

[1] Fallata E, Alamri AM, Alrabee HA, Alghamdi AA, Alsaearei A (2023). Chances of liver transplantation in a patient with transaldolase deficiency complicated by hepatopulmonary syndrome. Cureus.

[2] Tylki-Szymanska A, Wamelink MM, Stradomska TJ, Salomons GS, Taybert J, Dąbrowska-Leonik N, Rurarz M (2014). Clinical and molecular characteristics of two transaldolase-deficient patients. Eur J Pediatr.

[3] Banne E, Meiner V, Shaag A (2015). Transaldolase deficiency: a new case expands the phenotypic spectrum. JIMD Reports.

[4] Williams M, Valayannopoulos V, Altassan R (2019). Clinical, biochemical, and molecular overview of transaldolase deficiency and evaluation of the endocrine function: update of 34 patients. J Inherit Metab Dis.

[5] Xue J, Han J, Zhao X, Zhen L, Mei S, Hu Z, Li X (2021). Prenatal diagnosis of fetus with transaldolase deficiency identifies compound heterozygous variants: a case report. Front Genet.

[6] Takaleh A, Abunamous N, AlShamsi A, Alhassani N, Almazrouei R (2024). Hypergonadotropic hypogonadism due to transaldolase deficiency: two cases and literature review. JCEM Case Rep.

[7] Tylki-Szymańska A, Stradomska TJ, Wamelink MM, Salomons GS, Taybert J, Pawłowska J, Jakobs C (2009). Transaldolase deficiency in two new patients with a relative mild phenotype. Mol Genet Metab.

[8] Grammatikopoulos T, Hadzic N, Foskett P (2022). Liver disease and risk of hepatocellular carcinoma in children with mutations in TALDO1. Hepatol Commun.

[9] Rodan LH, Berry GT (2016). N-Acetylcysteine therapy in an infant with transaldolase deficiency is well tolerated and associated with normalization of alpha fetoprotein levels. JIMD Reports.

